# Adhesive-Free Cornhusk-Based Biocomposites via Alkali Retting–Pressing Strategy

**DOI:** 10.3390/polym18172176

**Published:** 2026-09-07

**Authors:** Rongbo Zheng, Kairui Zhang, Ning Xiao, Jiaofeng Fan, Xuelian Guo

**Affiliations:** 1 College of Biological & Chemical Engineering, Zhejiang University of Science and Technology, Hangzhou 310023, China; rbzheng@zust.edu.cn (R.Z.);; 2 Shaanxi Forest Products Quality Inspection and Industry Service Guarantee Center, Xi’an 710082, China

**Keywords:** cornhusk, biocomposites, higher performance, adhesive-free

## Abstract

Cornhusks are widely employed as reinforcing fillers in biocomposite fabrication, where conventional manufacturing processes rely on petroleum-derived resins or adhesives. This reliance not only poses substantial risks to human health and ecological environments, but also leads to the unsatisfactory mechanical performance of the final products. Developing a feasible strategy to eliminate petroleum-based binders while simultaneously enhancing the mechanical strength of the biocomposites remains a challenge. In this paper, we present an adhesive-free approach to produce high-performance, sustainable biomass structural materials directly from raw cornhusks, without prior pulverization, via a combined alkaline retting and hot-pressing treatment. Benefiting from the synergistic effects of highly aligned cellulose fibers and a densely compacted multi-layer structure, the resulting structural material exhibits a tensile strength of 136 MPa and a flexural strength of 127 MPa. These values are higher than those of conventional density fiberboards (approximately 20 MPa and 40 MPa). After 72 h of water immersion, the material shows a water absorption rate of 38% and a thickness swelling rate of 16%, both of which are lower than the corresponding parameters of traditional density fiberboards (around 60% and 20%). Its initial thermal degradation temperature reaches 251 °C, showing good thermal stability. Furthermore, the as-fabricated cornhusk-based structural material, which combines high mechanical strength, water resistance, and thermal resistance, exhibits zero formaldehyde emissions. It can serve as a promising alternative to traditional petroleum-bonded biomass density boards, for applications in furniture manufacturing and interior decoration.

## 1. Introduction

To effectively conserve wood resources, traditional biocomposites such as plywood, particleboard, and fiberboard have attracted attention for their applications in the household industry [1,2,3]. However, to manufacture high-performance biocomposites, synthetic adhesives including urea–formaldehyde or phenolic resins are widely used [4,5,6]. These adhesives not only accelerate the consumption of petroleum-based energy, but also release formaldehyde and cause environmental pollution. Researchers have devoted their efforts to developing green formaldehyde-free biocomposites [7,8,9]. For example, the lignocellulose fiber self-bonding strategy has been designed to prepare sustainable biocomposites via hot-pressing treatment using wood or bamboo fibers as raw materials [9,10,11]. Nevertheless, self-bonding biocomposites show poor mechanical properties, weak water resistance, and low thermal stability. Recently, Chen’s research group successfully fabricated bamboo self-bonding composites with superior water resistance and mechanical performance using high-consistency mechano-enzymatic (HCME) pretreated bamboo powders as raw materials [10]. HCME pretreatment facilitates bamboo-fiber defibrillation, generating subfibrous branches and fragmented parenchyma cells with increased specific surface areas to form a mechanical interlocking structure, which contributes to both mechanical reinforcement and improved water stability. Alternatively, chemical pretreatments such as alkali retting have been developed to enhance the performance of adhesive-free biocomposites [12,13,14]. Notably, the alkaline treatment of cornhusk can be carried out under milder conditions (i.e., room temperature, low concentration, and no Na_2_SO_3_ addition) compared with those for wood and bamboo [14]. Furthermore, an in situ regeneration and chemical delignification combined strategy has been designed to fabricate bioplastics and structural materials: lignocellulose is dissolved in deep eutectic solvents (DESs) or ionic liquids (ILs), followed by in situ regeneration and hot-pressing [15,16,17]. However, the requirement of DES or IL for lignocellulose dissolution leads to a complex process and high production cost. Therefore, it remains a challenge to produce high-performance biocomposites through a green, simple, and low-cost procedure.

Apart from wood and bamboo, natural cornhusk (NCH) has also been explored as a raw material for biocomposite fabrication. As an agricultural byproduct of corn—one of the most widely cultivated annual food crops worldwide—cornstalk, and corncob, NCH features an inherent 2D sheet structure with a thickness range of 0.1–1 mm, low cost, and abundant reserves. NCH has a high cellulose content of approximately 46% and a growth cycle of only several months. A total of 20 million tons of NCH can be generated annually in China, calculated based on the 7% mass ratio of cornhusk to corn kernel [18]. However, only a small fraction of NCH is currently utilized as animal feed or raw material for carpet and handbag weaving, while the majority is directly incinerated or discarded as waste, posing severe environmental pollution risks. Recent advances in related research have opened up new opportunities for the high-value utilization of NCH [19], including cellulose fibers [18], cellulose nanofibrils [20,21,22], hierarchical porous carbon [23], anthocyanin pigments [24], cornhusk cellulose-based films [25,26], cornhusk fiber-reinforced polyester/epoxy composites [27,28,29,30,31], and other NCH-derived functional products [32,33]. For NCH-based composites, NCH flour or fibers are commonly used as fillers to reinforce synthetic polymer matrices including polyethylene, polyester, and epoxy, as well as degradable or biomass-derived matrices such as starch, polylactic acid (PLA) and citric acid-based systems [27,28,29]. For instance, Youssef et al. adopted NCH powder as reinforcement to fabricate recycled low-density polyethylene composites, and the tensile strength of the composites was increased to 24.7 MPa [27]. In alternative routes, the whole NCH sheet without pulverization pretreatment has been directly utilized to prepare epoxy laminate composites [31], flame-retardant hydroxyapatite/nanoclay/MgSO_4_/NCH composites [34], transparent bioplastics [35,36], and even materials for solar-driven seawater desalination [37]. For instance, delignified NCH films have been developed as a matrix material to fabricate transparent fluorescent cornhusk materials for Fe^3+^ detection [36], unpowered thermoresponsive sensors [38], and flame-retardant cornhusk products [34]. Furthermore, our previous work demonstrated that NCH can be successfully converted into a transparent all-lignocellulosic bioplastic via a simple delignification–splicing strategy [35]. Notably, large-size glue-free cornhusk bioplastic sheets can be readily produced by mechanically pressing the overlapping edges of multiple delignified cornhusks arranged along the fiber orientation, which reveals the great application potential of delignified NCH for adhesive-free high-performance biocomposites [35].

In this paper, a robust and efficient micro/nanostructure design strategy is proposed to fabricate high-strength, sustainable cornhusk-derived structural materials via a simple two-step process: mild alkali delignification of NCH followed by direct hot-pressing. Benefiting from the synergistic effects of intrinsically oriented cellulose fibers, the preserved, natural multi-layer architecture, and the densely formed inter-molecular hydrogen-bonding network, the resultant structural material shows an high tensile strength of 136 MPa, a high flexural strength of 127 MPa, as well as good water resistance and thermal stability. Notably, no additional binders or synthetic polymers are introduced throughout the entire fabrication process, which greatly reduces the consumption of petrochemical resources and eliminates formaldehyde emissions during subsequent application. This NCH-derived biocomposite not only acts as a high-performance alternative for engineering structural uses, but also offers design insights for the micro/nanostructure construction of sustainable biocomposites.

## 2. Materials and Methods

Cornhusk, an agricultural waste product, was collected by local farmers and used as the starting material of cornhusk-derived structural materials (CHBCs). Sodium hydroxide (>98%) and ethanol (95%) were purchased from Sinopharm Chemical Reagent Co., Ltd. (Shanghai, China). All starting materials and reagents were used without further purification.

Cornhusks were cut into rectangular pieces of 4 × 8 cm^2^ or 10 × 10 cm^2^, then immersed in an aqueous solution of 4% (*w*/*w*) NaOH at room temperature for 3 h. Then, the swollen cornhusks were immersed in 44 wt% ethanol/water (1/1, *v*/*v*) at room temperature for 30 min to remove residual chemicals and to regenerate the dissolved lignocellulose in the porous structure of delignified cornhusks. This operation (washing with ethanol/water) was repeated three times. Subsequently, multiple pieces of swollen cornhusks were X-Y (90°) stacked and hot-pressed (180 °C) at a pressure of 10 MPa for 3 h. Finally, CHBC was obtained after natural cooling to room temperature with the pressure maintained.

The morphology of the samples was observed with a field-emission scanning-electron microscope (TESCAN, Brno-Kohoutovice, Czech Republic) under an accelerating voltage of 1 kV. All samples were gold sputtered with gold for 30 s at a constant current of 30 mA before observation. The cellulose, hemicellulose, and lignin contents of the samples were tested by using the standard methods of the STANDARD technical Association for the pulp and paper industry [14,39]. FT-IR spectra were acquired by a FT-IR spectrometer (Bruker Karlsruhe, Germany) over the range of 400–4000 cm^−1^ in attenuated-total-reflectance (ATR) mode. XRD patterns were collected using A Bruker D8 X-ray diffractometer (MTS, Shanghai, China) equipped with a curved detector manufactured at a scanning rate of 4° min^−1^ over the 2θ range of 10–45° by operating tube voltage of 40 kV, tube current at 30 mA, Cu Kαλ = 1.5406 Å [40]. TGA data were measured in air atmosphere with a TA Instruments SDT Q600 thermogravimetric analyzer (TA Instruments, New Castle, DE, USA) using a heating rate of 10 °C min^−1^ in air [9,41,42].

The density, tensile strength, and flexural strength of CHBCs were examined according to the national standard GB/T 17657-2013 [9,41]. Five measurements were recorded for each sample and the average values of the tensile strength, bending strength, and density were used. The mechanical performance of the samples was evaluated using a universal testing machine (ProLine Z020TN, Zwick, Ulm, Germany). The sample size was 50 × 8 × 2.5 mm^3^ (length × width × thickness), the upper- and lower-gauge distance of the tensile test was 17 mm, and the crosshead speed was 1.3 mm/min. The bending test adopted a three-point bending device with a span of 25.13 mm and a crosshead speed of 10 mm/min.

The water stability of the samples was evaluated by water contact angle and water uptake. Water contact angles on the sample surface were measured using a DSA 100 device (Krüss GmbH, Shanghai, China). Contact angles were determined using DSA 3 software [35,40]. Water uptake was determined by keeping the NCH and CHBC submerged under water for a total of 72 h. The specimens were withdrawn periodically from the water, blotted tightly with absorbent paper, and weighed. The percentage of weight change was used to determine water uptake.

## 3. Results and Discussion

**Figure 1:**
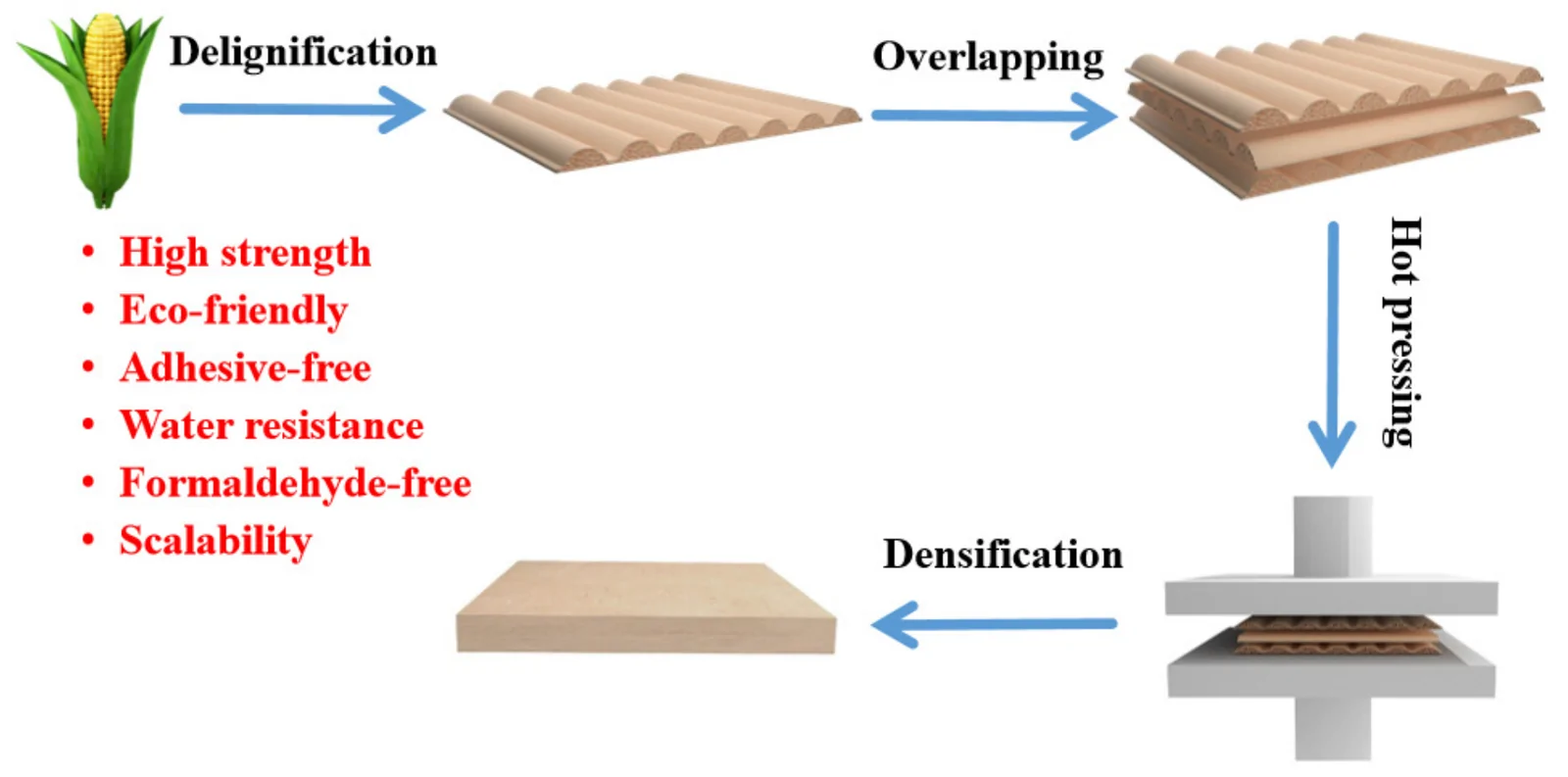
Graphic illustrating the preparation of CHBC from natural cornhusk (NCH).

We developed a simple, scalable, low-cost, adhesive-free approach that enables the direct fabrication of CHBC via an alkali-delignification and hot-pressing strategy. Figure 1 illustrates the fabrication schematic of this high-performance CHBC directly produced from discarded natural cornhusks (NCHs). The raw NCH possesses an inherent 2D layered structure consisting of vertically arranged microchannels and naturally distributed vascular bundles [42]. First, NCH is partially delignified in the NaOH aqueous solution at room temperature, which etches the surface of the raw material to expose the internal cellulose microfiber network. The delignified cornhusks are then rinsed with an ethanol–water mixed solution, followed by multi-layer stacking and lamination (Figures S1 and S2). Subsequent hot-pressing treatment promotes the thermal flow and redistribution of residual lignin, which enables the cellulose microfibers to be tightly and uniformly encapsulated by the lignin. The synergistic effect of heat and pressure during this process ensures a tight interfacial combination between cellulose and residual lignin, while each stacked cornhusk layer is bonded through abundant intermolecular hydrogen bonds and in situ regenerated lignocellulose. This endows the as-prepared CHBC with a highly densified internal structure and excellent mechanical strength, making it a promising alternative to medium-density fiberboard (MDF), high-density fiberboard (HDF), and even natural wood.

### 3.1. Appearance and Components of NCH and CHBC

**Figure 2:**
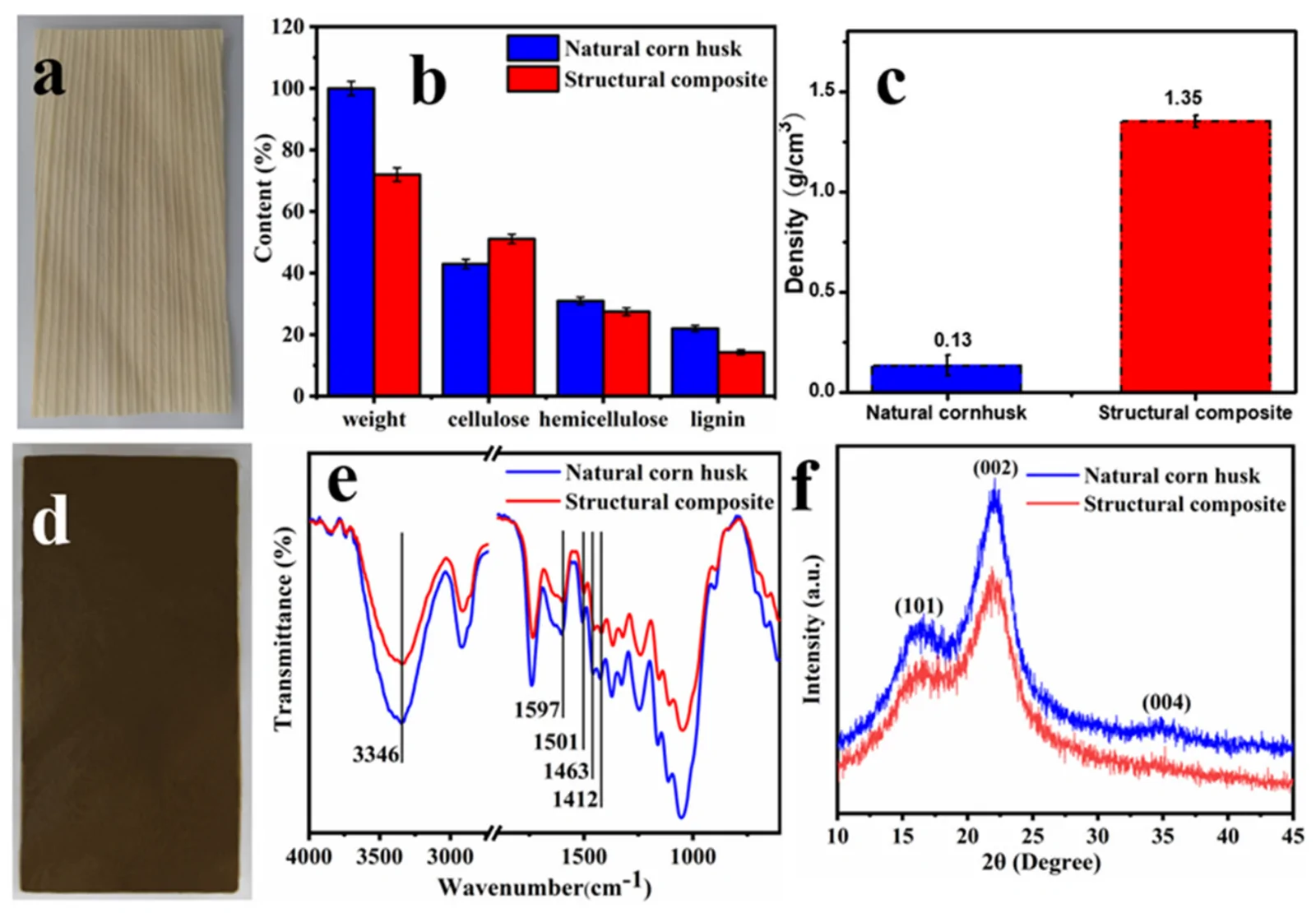
Comparison of components and other characteristics of NCH and CHBC. Photograph of NCH (**a**); changes in weight and cellulose, hemicellulose, and lignin content between NCH and CHBC (**b**); densities of NCH and CHBC (**c**); photograph of CHBC (**d**); FT-IR spectra (**e**) and XRD (**f**) of NCH and CHBC.

The appearance and chemical components’ evolution from NCH to the final biocomposites were characterized via Fourier transform infrared spectroscopy (FT-IR), X-ray diffraction (XRD), as well as the quantitative determination of hemicellulose/lignin content and bulk density. Owing to the existence of lignin, the raw NCH presents a pale-yellow appearance (Figure 2a), with naturally aligned vascular bundles distributed across the surface and distinct grooves formed between adjacent bundles. As shown in Figure 2b, the raw NCH is mainly composed of 43 wt% cellulose, 31 wt% hemicellulose, and 22 wt% lignin. After mild alkali-retting treatment, the obtained delignified cornhusk (DCH) turns into a lighter pale yellow, with significantly improved optical transmittance and excellent flexibility [14]. Subsequent mechanical hot-pressing induces a remarkable densification of the initial porous structure of NCH, leading to a sharp increase in bulk density from 0.13 g/cm^3^ for raw NCH to 1.35 g/cm^3^ for the final CHBC (Figure 2c). Compared with raw NCH, the CHBC shows a dark-brown color; a highly smooth, refined surface; and a dense structure (Figure 2d and Figure S3), which can be attributed to the melt flow of residual lignin under a high temperature and the complete elimination of internal pores during hot-pressing [43]. Correspondingly, the relative content of cellulose in CHBC increases obviously, while the absolute content of hemicellulose and lignin decreases slightly after partial removal during the alkali-retting process. As illustrated in Figure 2e, FT-IR spectra further reveal the chemical structure evolution throughout the fabrication process. The characteristic absorption peaks located at 1593, 1502, 1460, and 1426 cm^−1^, which are assigned to aromatic skeletal vibration, C-H vibration of methyl/methylene groups, and aromatic ring C-H vibration in lignin, show noticeable intensity weakening in the spectrum of CHBC, confirming that partial lignin is effectively removed during the alkali-retting process. Meanwhile, the characteristic peak at 1249 cm^−1^ corresponding to the -(C=O)-O- stretching vibration in hemicellulose also decreases, indicating the partial removal of hemicellulose during the alkali-retting process. The typical cellulose peaks at 1034 cm^−1^ and 1740 cm^−1^ maintain nearly identical intensity in the spectrum of the final CHBC. This phenomenon demonstrates that most of the cellulose samples are well-preserved after mild treatment. The XRD patterns presented in Figure 2f confirm that the difference in the crystalline structure of cellulose between raw NCH and CHBC is negligible. The distinct diffraction peaks corresponding to the (110), (020), and (040) crystal planes of cellulose I indicate that the native crystalline structure of cellulose is not damaged by the weak alkali swelling and subsequent hot-pressing treatment.

### 3.2. The Evolution of Morphology from NCH to CHBC

**Figure 3:**
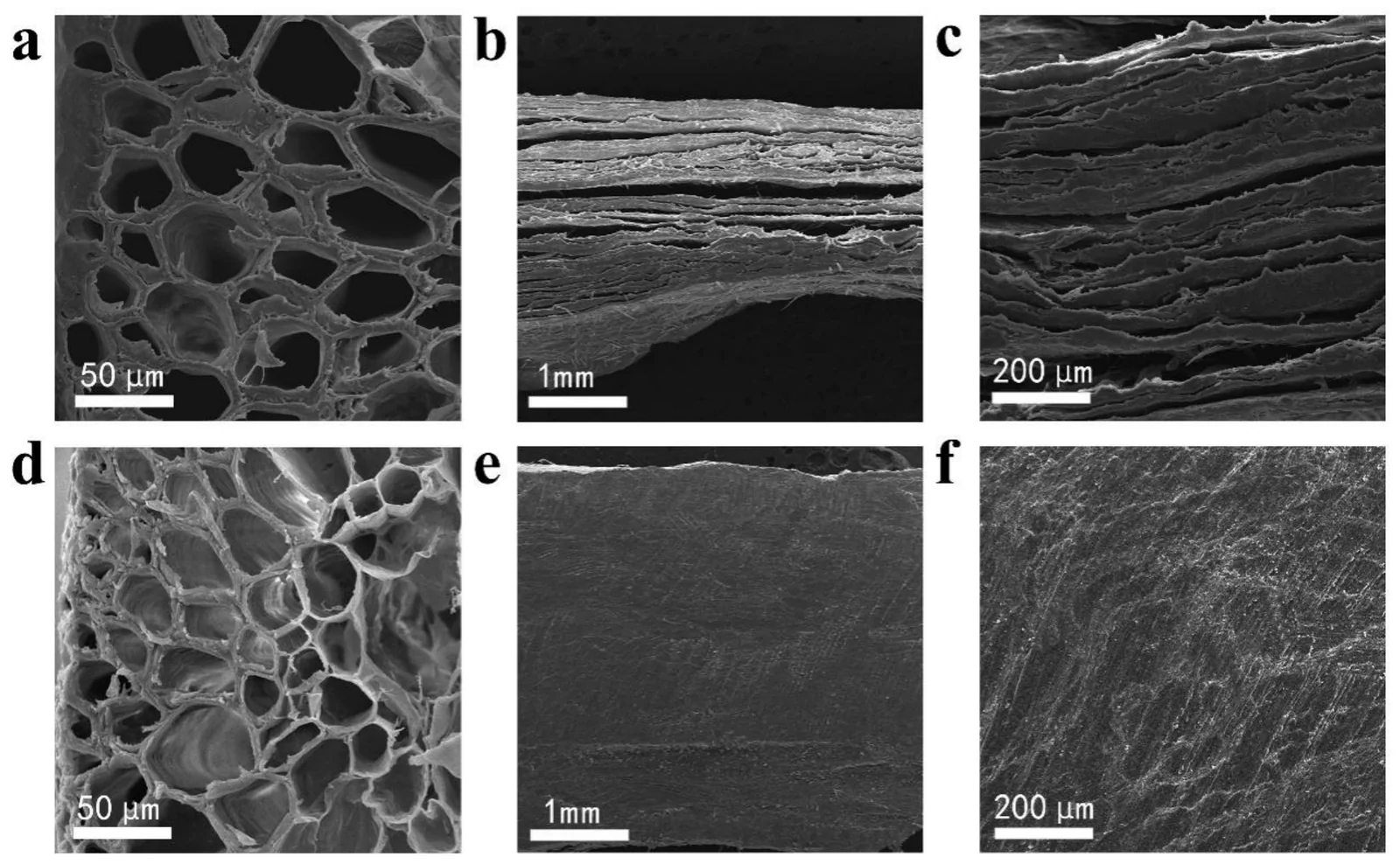
Cross-sectional SEM images of NCH (**a**), hot-pressed NCH (**b**,**c**), DCH (**d**), and CHBC (**e**,**f**).

NCH inherently possesses a porous structure assembled by vessel elements, parenchyma cells, vascular bundles, and interconnected tubular channels. The cross-sectional scanning electron microscopy (SEM) image of NCH reveals a well-aligned porous structure with an irregular honeycomb-like morphology (Figure 3a). When NCH is directly hot-pressed without alkali retting, distinct interlayer gaps can be clearly observed, which indicates insufficient densification caused by the rigid, native, porous structure of NCH (Figure 3b,c). After mild alkali retting at room temperature, the obtained DCH still retains its initial 3D porous framework, including the long tubular channels, parenchyma cells, and vascular bundles [14]. Meanwhile, the DCH becomes significantly swollen and exhibits a rougher surface compared with the raw NCH, and the thickness of its cell wall is remarkably reduced (Figure 3d) [35]. This morphological change mainly originates from the partial removal of lignin, which exposes more internal cellulose surfaces and greatly facilitates the subsequent hot-pressing-induced densification process. During the hot-pressing procedure, precisely controlling the temperature to trigger the melt flow of residual lignin while preserving the intact native cellulose structure is a critical factor for enhancing the mechanical performance of the final biocomposites. Previous studies have reported that the glass transition temperature of lignin ranges from 100 to 130 °C, while cellulose only melts at 467 °C in pulsed thermal tests [43,44]. Within the selected temperature window range of 100~130 °C, residual lignin undergoes thermal melting and uniformly disperses into the cellulose matrix, without causing any structural damage to cellulose. Therefore, when multi-layer DCH sheets are hot-pressed at 180 °C, the original tubular channels and cell walls collapse completely to form a fully densified architecture, and adjacent cornhusk layers achieve seamless interfacial bonding, as directly verified by the cross-sectional SEM images of the final CHBC (Figure 3e,f).

### 3.3. Mechanical Strength of Natural Wood and CHBC

**Figure 4:**
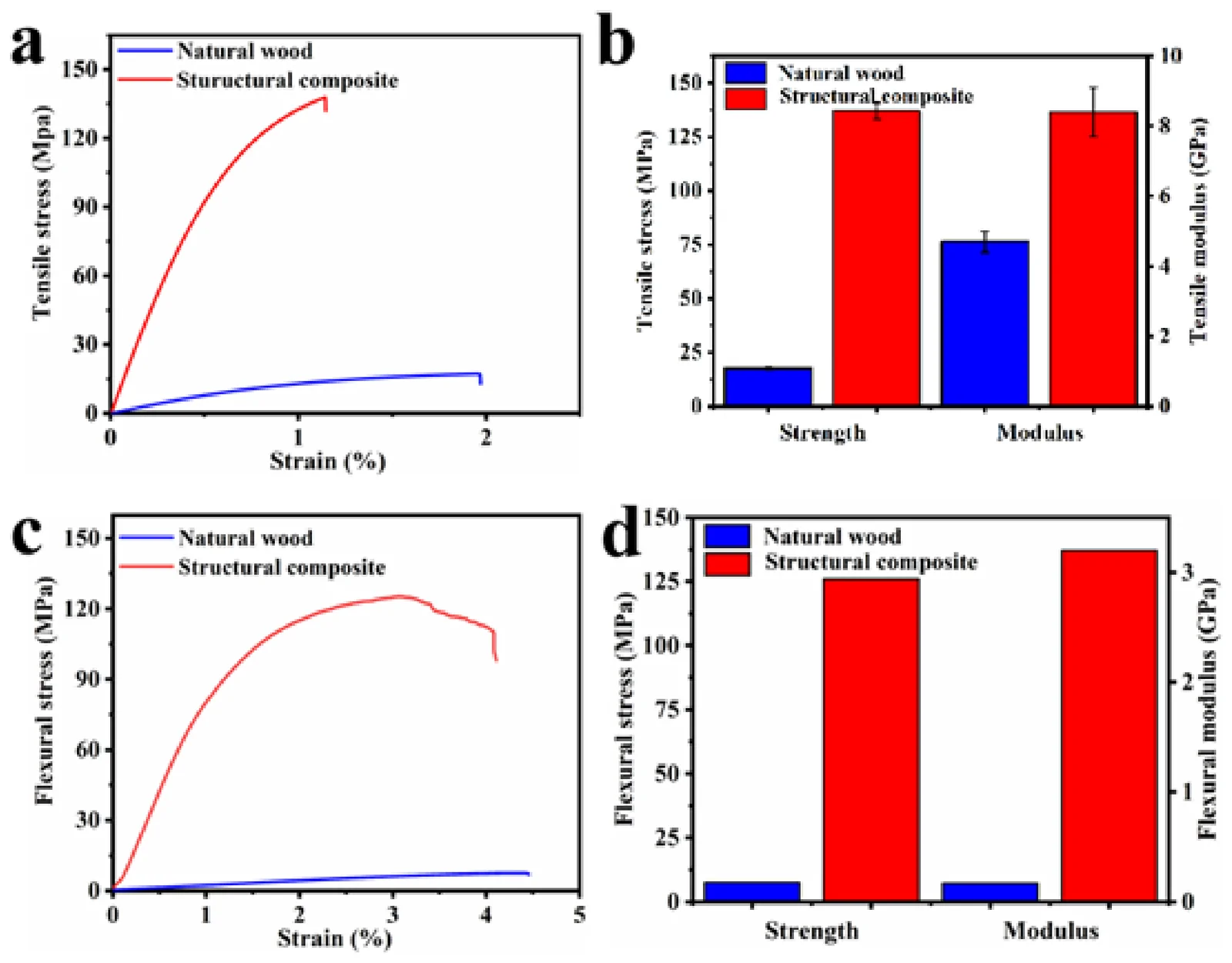
Mechanical properties of natural wood and CHBC. Tensile strength (**a**) and tensile strength comparison (**b**) for natural balsa wood and CHBC; flexural strength (**c**) and flexural strength comparison (**d**) for natural wood and CHBC.

Mechanical performance is an important indicator that determines the service reliability of structural materials. Higher mechanical strength enables the material to maintain structural integrity under external mechanical impacts. For NCH, the tensile strength along the fiber-growth direction (L) is significantly higher than that perpendicular to the growth direction (T) (Figure S4), and the mechanical strength in both directions is far lower than that of most natural wood species. This obvious tensile anisotropy originates from the unidirectionally oriented cellulose structure and the high inherent porosity of NCH, which severely limits its practical applications in complex load-bearing scenarios. After partial alkali delignification followed by hot-pressing-induced full densification, the as-prepared CHBC forms a highly homogeneous internal structure, which endows the material with nearly identical tensile strength in both in-plane directions. The mechanical properties of the structural composite were further quantitatively evaluated via uniaxial tensile tests. The CHBC exhibits a maximum tensile strength of 136 MPa and a Young’s modulus of 8.4 GPa, which is more than five-times higher than that of the raw untreated NCH (Figure 4a,b). The enhancement in tensile strength clearly demonstrates that the partial delignification and subsequent densification processes are highly effective strategies for reinforcing the mechanical performance of the biocomposite. Specifically, the partial removal of amorphous lignin exposes a large number of active hydroxyl groups on the cellulose surface, which significantly promotes the formation of dense intermolecular hydrogen bonds and greatly improves the interfacial interaction between adjacent cellulose microfibers [45]. Meanwhile, the residual lignin undergoes thermal softening during the hot-pressing process and uniformly disperses into the cellulose matrix [9,43]. The abundant hydroxyl groups in the residual lignin structure further participate in the construction of a robust, physical cross-linking network through intermolecular hydrogen bonding with cellulose chains. Moreover, the high, native orientation of cellulose nanofibrils inherited from raw NCH is well-preserved in the final CHBC, forming a rigid and stable cellulose skeleton that further contributes to the mechanical performance. As shown in Figure 4c,d, the flexural strength of CHBC is also higher than that of natural wood.

To comprehensively assess the structural performance of the developed CHBC, a systematic comparison was conducted against typical natural wood species and commercially available wood-based panels. The tensile strength (136 MPa) of CHBC exceed that of fiberboard (e.g., MDF, ~17 MPa; HDF, ~19 MPa), common softwoods (e.g., pine, ~70 MPa; cedar, ~46 MPa), and most hardwoods (e.g., poplar, ~50 MPa; oak, ~115 MPa) [9,12,39]. Moreover, CHBC exhibits a density (1.35 g/cm^3^) higher than that of fiberboard (MDF, ~0.7–0.9 g/cm^3^; HDF, ~0.9–1.2 g/cm^3^). As for the bending strength, CHBC reaches 127 MPa, which is also higher than that of fiberboard (MDF, ~39 MPa; HDF, ~42 MPa), biocomposites (~50 MPa), and natural wood (e.g., poplar, ~75 MPa). More importantly, unlike natural wood, which often suffers from significant strength anisotropy due to its inherent structure, CHBC exhibits excellent isotropic mechanical properties with nearly identical tensile strength values in both in-plane directions.

### 3.4. Heat Resistance and Water Resistance

**Figure 5:**
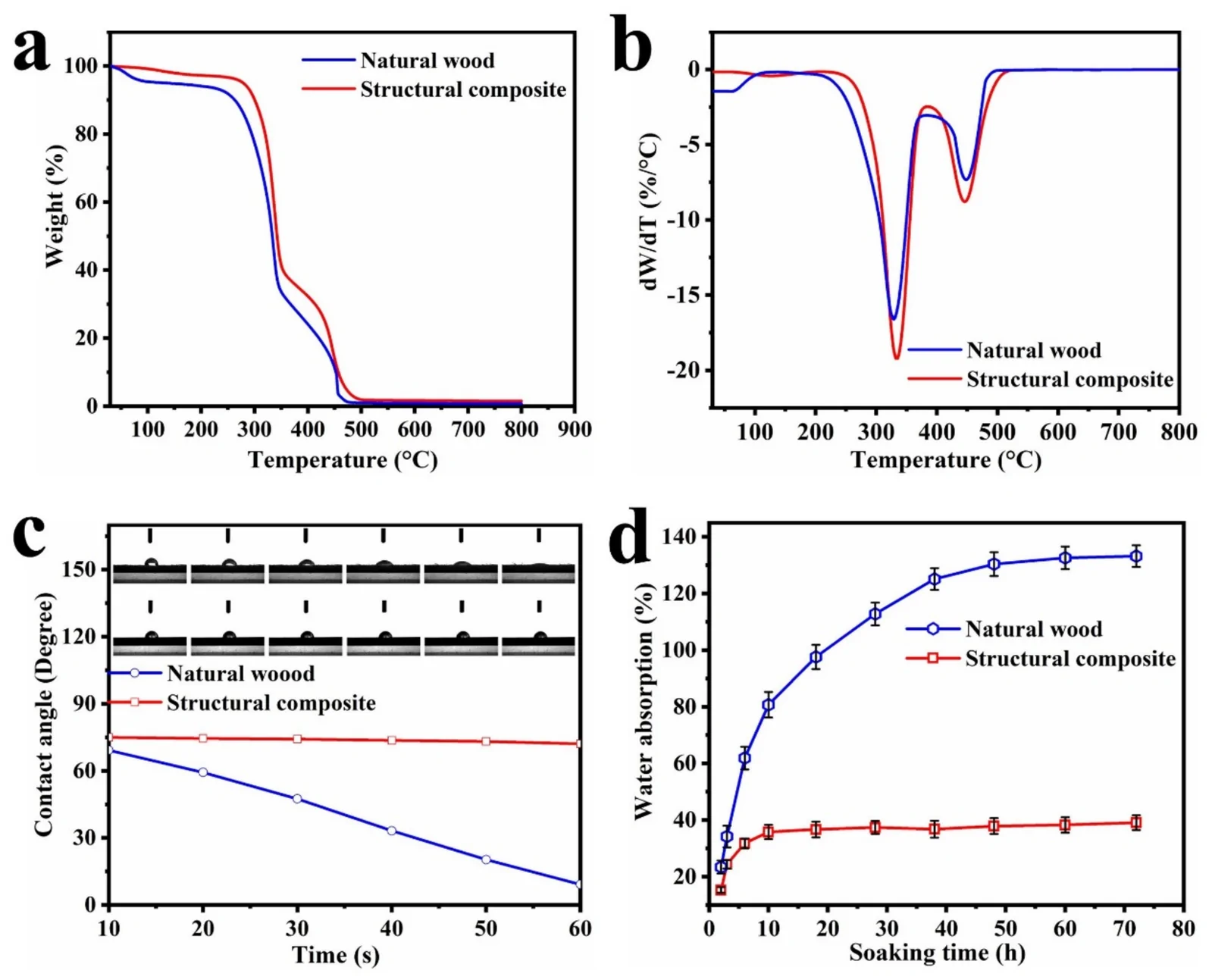
Thermal and water stability of natural wood and CHBC. TG (**a**), DTG (**b**), water contact angle (**c**), and water absorption (**d**) of natural wood and CHBC.

Thermogravimetric analysis (TGA) was employed to evaluate the thermal stability and degradation resistance of the CHBC in comparison with natural wood. As shown in Figure 5a, both materials exhibit three distinct mass-loss stages during pyrolysis in air, corresponding to dehydration, oxidative pyrolysis of main components, and subsequent coke oxidation [23]. The first stage (up to ~100 °C) is attributed to the evaporation of physically absorbed moisture. For natural wood, a clear mass loss is observed in this region, whereas the TGA curve of CHBC remains nearly flat, which can be ascribed to the prior hot-pressing treatment at 180 °C that effectively removes most free water. The second stage, representing the primary decomposition of hemicellulose and cellulose, starts at 222 °C for natural wood and continues until 369 °C. In contrast, CHBC exhibits a significantly delayed onset of decomposition at 251 °C, with the major mass loss extending to 380 °C. The delay of the onset temperature directly demonstrates the good thermal stability of CHBC, which originates from the partial removal of hemicellulose during the alkali-retting process and the formation of a denser, more thermally resistant structure. The third stage corresponds to the further oxidation of the remaining char. CHBC begins this stage at approximately 400 °C and completes it by 499 °C, which occurs slightly earlier than that of natural wood (430–481 °C). This phenomenon can be explained by the reduced lignin content in CHBC. Since lignin undergoes decomposition across a wide temperature span (typically the range of 195–420 °C), partial delignification results in a relatively earlier and more pronounced final decomposition step for CHBC. Overall, CHBC demonstrates enhanced thermal stability and degradation resistance during combustion compared to natural wood. This improvement is attributed to its densified microstructure, the partial removal of thermally less-stable hemicellulose, and the retained lignin that promotes the formation of a protective carbonized layer around the cellulose fibers, thereby delaying heat transfer and mass loss.

The inherent densified structure and natural hydrophobicity of lignin contribute significantly to maintaining the dimensional and structural stability of CHBC under humid conditions. Contact angle measurements reveal that the initial water contact angle of the CHBC surface is approximately 75°, which is higher than that of natural wood (~65°). Owing to the strong hydrophilicity of cellulose and its loose, porous structure, the water contact angle of natural wood rapidly decreases to 0° within a short period. In contrast, the water contact angle of CHBC remains stable at around 63° even after 60 s (Figure 5c), indicating its superior surface water resistance and stability. Long-term immersion tests further demonstrate the exceptional water resistance of CHBC. After 72 h of water immersion, the water absorption rate of natural wood exceeds 100%, whereas that of CHBC stabilizes at only about 38% (Figure 5d). This value is also lower than that of commercial MDF, HDF, and poplar, which typically exhibit water absorption rates around the 60–70% range [9,43]. Moreover, the CHBC sample maintains its original shape without any noticeable swelling, deformation, or structural disintegration after prolonged water exposure. The enhanced water stability of CHBC can be attributed to the following factors. First, lignin possesses a hydrophobic phenylpropane skeleton, which provides innate water-repellent properties. During hot-pressing, the molten lignin flows and uniformly coats the cellulose microfibrils, forming a protective hydrophobic layer that shields the hydrophilic cellulose from direct water contact. Second, the hot-pressing process thoroughly eliminates the inherent micropores and tubular channels within the raw cornhusk, resulting in a highly densified structure that significantly reduces water infiltration pathways. Third, the partial removal of hemicellulose—a highly hydrophilic component—further diminishes the material’s overall affinity for moisture. Consequently, CHBC exhibits excellent water resistance, making it suitable for applications in humid or even harsh environments where conventional wood-based materials would undergo severe swelling.

### 3.5. Advantages and Prospects of CHBC

Our alkali-retting and hot-pressing strategy for fabricating CHBC exhibits several distinctive advantages over conventional methods for producing cornhusk-based structural composites. This approach is more environmentally benign, green, adhesive-free, sustainable, requires less chemical/energy input, and possesses a lower carbon footprint. Conventional methods typically involve pulverizing cornhusk into particles or short fibers to serve as fillers, which are then combined with synthetic polymer matrices (e.g., epoxy resin) or formaldehyde-based adhesives. In contrast, our process employs mild alkali delignification at room temperature, using only NaOH or KOH, water, and raw cornhusk as starting materials, eliminating the need for any external binders or hazardous chemicals. The as-prepared CHBC demonstrates comprehensively enhanced properties. It exhibits a higher mechanical strength and modulus, better water resistance, and improved thermal stability compared to commercial wood-based panels such as MDF, HDF, and even natural wood. Since no formaldehyde or other volatile organic compound (VOC)-releasing adhesives are used, CHBC is inherently formaldehyde-free. This feature makes it particularly suitable for indoor applications, such as furniture, decorative panels, and children’s products, where indoor air quality and human health are major concerns. Based on a process yield of approximately 74% [14], we estimate that about 14.6 million tons (equivalent to ~11 million m^3^) of CHBC could be produced annually from the cornhusk waste generated in China. This volume represents roughly 11% of China’s annual timber imports, highlighting its substantial potential for supplementing and partially replacing traditional wood resources. The estimated production cost of CHBC is approximately USD 260/m^3^. This unit cost is lower than that of pine panels used for furniture and decoration (400–600/m^3^) and is comparable to that of standard biomass particleboards (E1/ENF grade, USD 200–380/m^3^), indicating economic competitiveness. The CHBC developed via our simple and scalable strategy presents a promising, high-performance, and sustainable alternative to conventional wood-based panels and petroleum-derived composites, with clear advantages in environmental friendliness, performance, cost, and carbon balance.

## 4. Conclusions

In summary, this work presents a robust and effective micro/nanostructure design strategy to directly convert NCH into high-performance, sustainable, structural biocomposites. The synergistic effects of cross-laminated architecture, highly aligned cellulose fibers, and dense hydrogen-bonding networks are systematically elucidated. The resultant CHBC achieves a tensile strength of 136 MPa, a flexural strength of 127 MPa, excellent water resistance, and enhanced thermal stability. The obtained CHBC not only matches, but surpasses natural wood in key mechanical metrics. Furthermore, the entire fabrication process is binder- and polymer-free, significantly reducing energy and time consumption compared to conventional composite manufacturing. Collectively, these findings provide innovative guidance for the design of binder-free, sustainable, and environmentally benign biomass-based structural materials. They also demonstrate a viable pathway for valorizing abundant agricultural residues, thereby expanding the potential for their application in next-generation green construction, furniture, and engineered biocomposites.

## Data Availability

Data are contained within the article.

## Supplementary Materials

The following supporting information can be downloaded at: https://www.mdpi.com/article/10.3390/polym18172176/s1, Figure S1: photo of delignified cornhusk; Figure S2: photos of overlapped delignified cornhusks; Figure S3: cross-sectional photo of CHBC; Figure S4: tensile strength of NCH; Table S1: the material price of NCH.

## References

[1] Sampathrajan A., Vijayaraghavan N.C., Swaminathan K.R. (1992). Mechanical and thermal properties of particleboards made from farm residues. Bioresour. Technol..

[2] Li X., Li Y.H., Zhong Z.K., Wang D., Ratto J.A., Sheng K., Sun X.S. (2009). Mechanical and water soaking properties of medium density fiberboard with wood fiber and soybean protein adhesive. Bioresour. Technol..

[3] Khalil H.P.S.A., Fazita M.R.N., Bhat A.H., Jawaid M., Fuad N.N. (2010). Development and material properties of new hybrid plywood from oil palm biomass. Mater. Des..

[4] Halvarsson S., Edlund H., Norgren M. (2008). Properties of medium-density fibreboard (MDF) based on wheat straw and melamine modified urea formaldehyde (UMF) resin. Ind. Crops Prod..

[5] Mo X., Cheng E., Wang D., Sun X. (2003). Physical properties of medium-density wheat straw particleboard using different adhesives. Ind. Crops Prod..

[6] Khalil H.P.S.A., Firdaus M.Y.N., Jawaid M., Anis M., Ridzuan R., Mohamed A. (2010). Development and material properties of new hybrid medium density fibreboard from empty fruit bunch and rubberwood. Mater. Des..

[7] Saenghirunwattana P., Noomhorm A., Rungsardthong V. (2014). Mechanical properties of soy protein based “green” composites reinforced with surface modified cornhusk fiber. Ind. Crops Prod..

[8] Nakanishi E.Y., Cabral M.R.C., Gonçalves P.S., dos Santos V., Junior H.S. (2018). Formaldehyde-free particleboards using natural latex as the polymeric binder. J. Clean. Prod..

[9] Ye H., Wang Y., Yu Q., Ge S., Fan W., Zhang M., Huang Z., Manzo M., Cai L., Wang L. (2022). Bio-based composites fabricated from wood fibers through self-bonding technology. Chemosphere.

[10] Cheng P., Zhong T.H., Liu X., Chen H. (2025). Bamboo self-bonding composites with superior water resistance and mechanical performance prepared with high-consistency mechano-enzymatic pretreated bamboo powders. ACS Sustain. Chem. Eng..

[11] Chen H., Shi J.J., Zhong T.H., Fei B., Xu X., Wu J., Shang L., Zhang W., Yuan S., Xia C. (2023). Tunable physical-mechanical properties of eco-friendly and sustainable processing bamboo self-bonding composites by adjusting parenchyma cell content. ACS Sustain. Chem. Eng..

[12] Song J.W., Chen C.J., Zhu S.Z., Zhu M., Dai J., Ray U., Li Y., Kuang Y., Li Y., Quispe N. (2018). Processing bulk natural wood into a high-performance structural material. Nature.

[13] Li Z.H., Chen C.J., Mi R.Y., Gan W., Dai J., Jiao M., Xie H., Yao Y., Xiao S., Hu L. (2020). A Strong, tough, and scalable structural material, from fast-growing bamboo. Adv. Mater..

[14] Zheng M., Zhang K.R., Zhang J., Zhu L., Du G., Zheng R. (2022). Cheap, high yield, and strong cornhusk-based textile bio-fibers with low carbon footprint via green alkali retting-splicing-twisting strategy. Ind. Crops Prod..

[15] Xia Q.Q., Chen C.J., Yao Y.G., Li J., He S., Zhou Y., Li T., Pan X., Yao Y., Hu L. (2021). A strong, biodegradable and recyclable lignocellulosic bioplastic. Nat. Sustain..

[16] Dong X.F., Gan W.T., Shang Y., Tang J., Wang Y., Cao Z., Xie Y., Liu J., Bai L., Li J. (2022). Low-value wood for sustainable high-performance structural materials. Nat. Sustain..

[17] Sun Y., Liu Y., Bai L., Liu S., Wan Z., Yang Z., Yu L., Huan S., Wang C., Li Z. (2026). Wood that bonds itself via homologous adhesion through cellulose reconstitution. Nat. Comm..

[18] Reddy N., Yang Y. (2005). Properties and potential applications of natural cellulose fibers from cornhusks. Green Chem..

[19] Ratna A.S., Ghosh A., Mukhopadhyay S. (2022). Advances and prospects of cornhusk as a sustainable material in composites and other technical applications. J. Clean. Prod..

[20] Mendes C.A.D.C., Ferreira N.M.S., Furtado C.R.G., de Sousa A.M.F. (2015). Isolation and characterization of nanocrystalline cellulose from cornhusk. Mater. Lett..

[21] Yang X., Han F., Xu C., Jiang S., Huang L., Liu L., Xia Z. (2017). Effects of preparation methods on the morphology and properties of nanocellulose (NC) extracted from cornhusk. Ind. Crops Prod..

[22] Valdebenito F., Pereira M., Ciudad G., Azocar L., Briones R., Chinga-Carrasco G. (2017). On the nanofibrillation of cornhusks and oat hulls fibres. Ind. Crops Prod..

[23] Song S., Ma F., Wu G., Ma D., Geng W., Wan J. (2015). Facile self-templating large scale preparation of biomass-derived 3D hierarchical porous carbon for advanced supercapacitors. J. Mater. Chem. A.

[24] Li C.Y., Kim H.W., Won S.R., Min H.-K., Park K.-J., Park J.-Y., Ahn M.-S., Rhee H.-I. (2008). cornhusk as a potential source of anthocyanins. J. Agric. Food Chem..

[25] Bernhardt D.C., Perez C.D., Fissore E.N., De’nObili M.D., Rojas A.M. (2017). Pectin-based composite film: Effect of cornhusk fiber concentration on their properties. Carbohydr. Polym..

[26] Chen Q.F., Xiong J.Y., Chen G.X., Tan T. (2020). Preparation and characterization of highly transparent hydrophobic nanocellulose film using cornhusks as main material. Int. J. Biol. Macromol..

[27] Youssef A.M., El-Gendy A.A., Kamel S. (2015). Evaluation of cornhusk fibers reinforced recycled low density polyethylene composites. Mater. Chem. Phys..

[28] Sari N., Prunch C., Sapuan S. (2020). The effect of water immersion and fibre content on properties of cornhusk fibres reinforced thermoset polyester composite. Polym. Test..

[29] Sari N., Suteja S., Fudholi A., Zamzuriadi A., Sulistyowati E.D., Pandiatmi P., Sinarep S., Zainuri A. (2021). Morphology and mechanical properties of coconut shell powder-filled untreated cornhusk fibre-unsaturated polyester composites. Polymer.

[30] Sari N.H., Suteja S., Fudholi A., Sutaryono Y.A., Maskur M., Srisuk R., Rangappa S.M., Siengchin S. (2022). Evaluation of impact, thermo-physical properties, and morphology of cornhusk fiber-reinforced polyester composites. Polym. Compos..

[31] Singh H., Chatterjee A. (2020). Potential of alkali treated cornhusk film as reinforcement for epoxy laminate composites. Cellulose.

[32] Wu M.X., Zhu Q.L., Ma D.L. (2025). Ni_3_V_2_O_8_@3D porous graphitic biogenic carbon composite electrode material for high performance supercapacitors. ACS Sustain. Resour. Manag..

[33] Zheng R.B., Zheng M., Guo X.L., Wang X., Liu X., Yan C. (2026). From spontaneously tubulate cornhusk to cheap, scalable, degradable biomass straws via adhesion of in-situ regenerated lignocellulose. ACS Sustain. Resour. Manag..

[34] Mehmood S., Wang J.Q., Shang Y.L., Lin C., Qiu W., Ran D. (2025). A green delignification and enhanced fire-retardancy of cornhusks for fire-safety applications. Sustain. Mater. Technol..

[35] Zhang K.R., Zhu L.L., Li H.Y., Zheng M., Zhang J., Zheng Y., Zheng R. (2022). From cornhusks to scalable, strong, transparent bio-plastic using direct delignification-splicing strategy. Adv. Sustain. Syst..

[36] Wu X.X., Mei X., Yuan X.S., Liu L., Guo H., Xie L., Chai X., Xu K., Du G., Zhang L. (2024). Preparation of high intensity transparent fluorescent cornhusk and its detection of Fe^3+^. Ind. Crops Prod..

[37] Mehmood S., Wang J.Q., Jiang J.L., Lin C., Shang Y., Ran D. (2025). Harvesting low-grade heat by architecting the hierarchical porous structure of green delignified cornhusk for solar-driven seawater desalination. J. Environ. Chem. Eng..

[38] Mehmood S., Wang J.Q., Li Z.M., Qiu W., Arshad A., Shang Y., Yang B., Zhu J., Shao Z., Mahmood H. (2024). A green approach for delignification of cornhusks and their application as an unpowered thermo-responsive sensor. Ind. Crops Prod..

[39] Li H.Y., Guo X.L., He Y.M., Zheng R. (2019). A green steam-modified delignification method to prepare low-lignin delignified wood for thick, large highly transparent wood composites. J. Mater. Res..

[40] Zhu L.L., Dang B., Zhang K.R., Zhang J., Zheng M., Zhang N., Du G., Chen Z., Zheng R. (2022). Transparent bioplastics from super-low lignin wood with abundant hydrophobic cellulose crystals. ACS Sustain. Chem. Eng..

[41] Yi X.Y., Zhang Z.X., Niu J.F., Wang H., Li T., Gong J., Zheng R. (2024). Green strong corstalk rind-based cellulose-PVA aerogel for oil adsorption and thermal insulation. Polymers.

[42] Zheng R.B., Fan J.F., Guo X.L., Liu X., Yan C. (2026). From self-tubulating cornhusk to green, cheap, high-performance biomass straws. Ind. Crops Prod..

[43] Shi Y., Cai Y.C., Jiang J.X., Ge S., Xi G., Xu B.B., Li J. (2025). Recycling waste lignin as natural adhesive to prepare sustainable wooden composite materials. EcoMat.

[44] Wang X., Xia Q., Jing S., Li C., Chen Q., Chen B., Pang Z., Jiang B., Gan W., Chen G. (2021). Strong, hydrostable, and degradable straws based on cellulose-lignin reinforced composites. Small.

[45] Zhu L.L., Chen T.A., Zheng Y.J., Zhang K.R., Zhang J., Zheng M., Zheng R.B. (2022). Preparation and enhanced photostability of TiO_2_@SiO_2_-delignified wood. Sci. Silvae Sin..

