# Supplementary material for: Adhesive-Free Cornhusk-Based Biocomposites via Alkali Retting–Pressing Strategy
Source: Polymers (Basel). 2026 Sep 7;18(17):2176. doi: 10.3390/polym18172176 (PMC13568321; doi:10.3390/polym18172176)
Supplement: Supplementary file 1 [file polymers-18-02176-s001.zip › polymers-4516251-supplementary.pdf]

Supporting Information

Adhesive-free cornhusk-based biocomposites via alkali retting-pressing strategy

Rongbo Zheng[1], Kairui Zhang[2], Ning Xiao[2], Jiaofeng Fan[1], Xuelian Guo[1]*

1 College of Biological & Chemical Engineering, Zhejiang University of Science and Technology, Hangzhou 310023, China.

2 Shaanxi Forest Products Quality Inspection and Industry Service Guarantee Center, Xi'an 710082, China.

*Corresponding author. E-mail: guoxuelian2023@zust.edu.cn

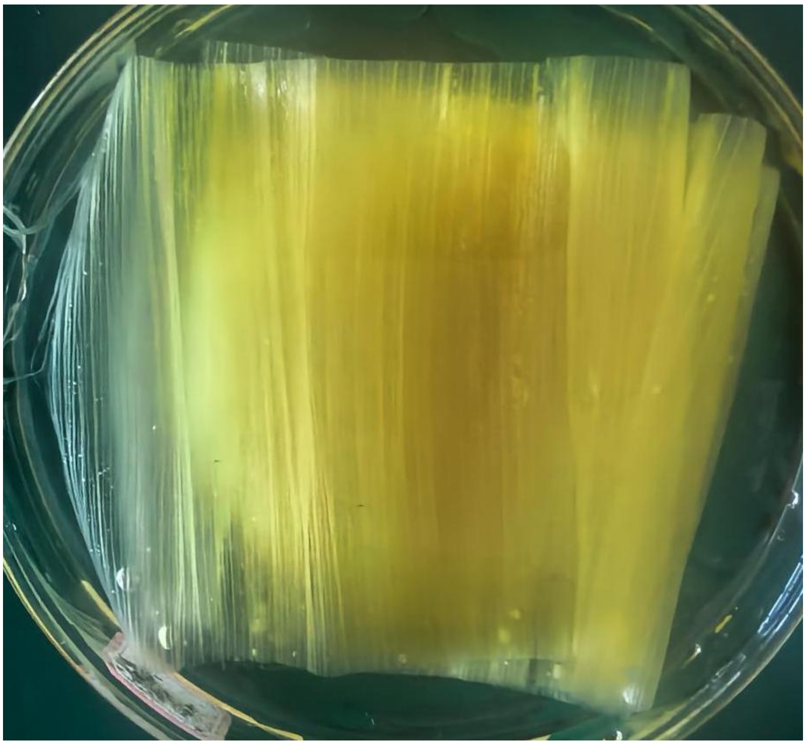

Figure S 1. Photo of delignified cornhusk.

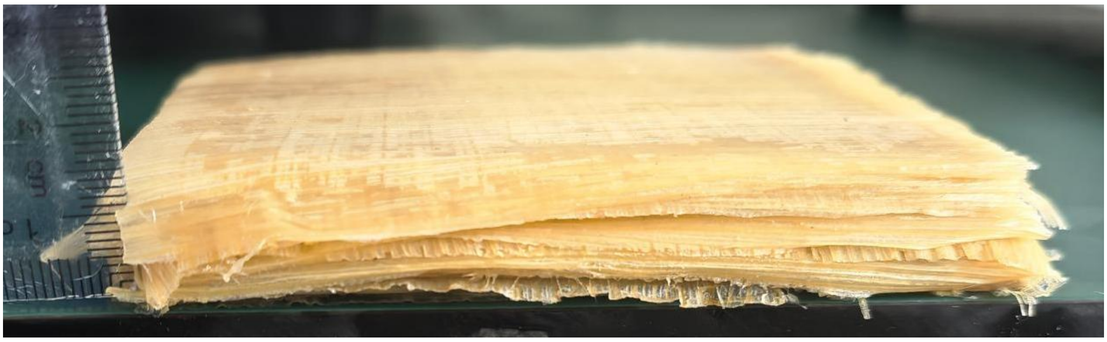

Figure S 2. Photo of overlapped delignified cornhusks.

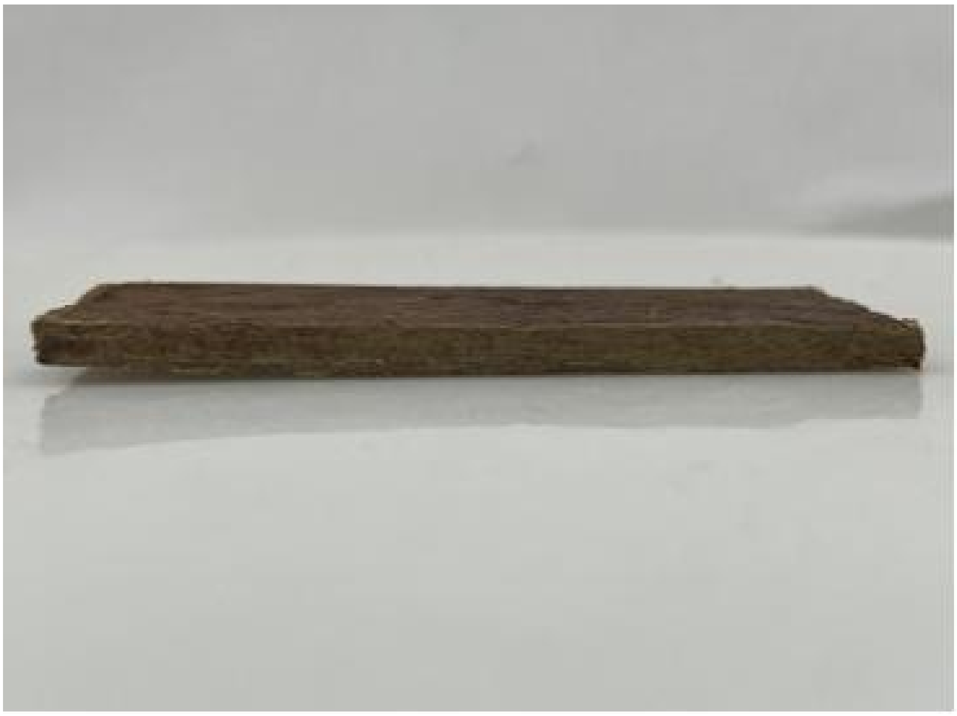

Figure S 3. Cross-sectional photo of CHBC.

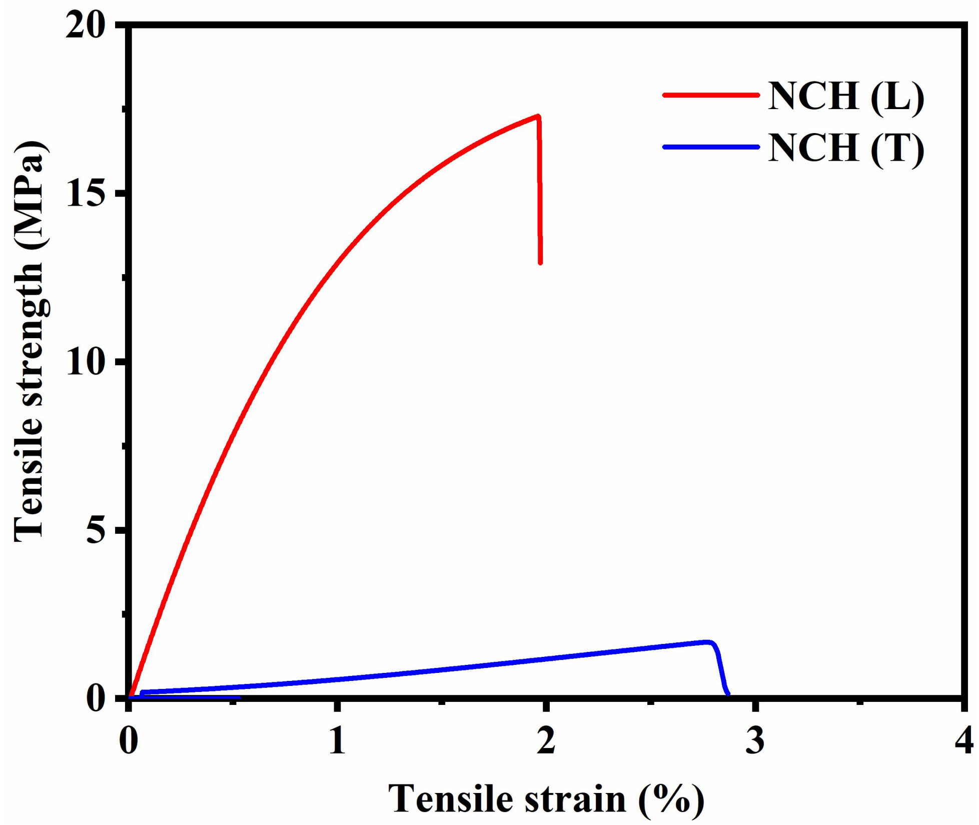

Figure S 4. Tensile strength of NCH.

Table S 1. The material price of NCH

Raw material and processing cost	Approximate usage mass (g/per straw)	Unite price	Approximate price ($/m^2)
NaOH	1.12 kg	$0.3 per kg	0.34
Cornhusk	9.3 kg	$0.1 per kg	0.93
Electricity	0.5/kWh/m^2	$0.093/kWh/20L	0.05
Aggregate	-		1.32

Note: 1. The thickness of structure materials is 5mm.

2. The mass ratio of cornhusk to NaOH aqueous solution (4 wt%) is 1:3
